# Disease Reactivation after Fingolimod Discontinuation in Pregnant Multiple Sclerosis Patients

**DOI:** 10.1007/s13311-021-01106-6

**Published:** 2021-09-07

**Authors:** Assunta Bianco, Matteo Lucchini, Rocco Totaro, Roberta Fantozzi, Giovanna De Luca, Sonia Di Lemme, Giorgia Presicce, Luana Evangelista, Valeria Di Tommaso, Roberta Pastorino, Chiara De Fino, Valeria De Arcangelis, Diego Centonze, Massimiliano Mirabella

**Affiliations:** 1 Multiple Sclerosis Center, Fondazione Policlinico Universitario Agostino Gemelli IRCCS, 00168 Rome, Italy; 2 Centro Di Ricerca Per La Sclerosi Multipla (CERSM), Università Cattolica del Sacro Cuore, 00168 Rome, Italy; 3 Demyelinating Disease Center, San Salvatore Hospital, L’Aquila, Italy; 4 Neurology Unit, IRCCS Neuromed, Pozzilli, IS Italy; 5 Multiple Sclerosis Centre, Unit of Neurology, SS Annunziata University Hospital, Chieti, Italy; 6 Department of Woman and Child Health and Public Health-Public Health Area, Fondazione Policlinico Universitario A. Gemelli IRCCS, 00168 Rome, Italy; 7 Department of Systems Medicine, Tor Vergata University, Rome, Italy

**Keywords:** Pregnancy, Delivery, Rebound, Breastfeeding, Relapse

## Abstract

Recent studies estimated an incidence of 4–25% of disease rebound after withdrawal of fingolimod (FTY) for any reason, but specific data on disease reactivation after FTY withdrawal due to pregnancy are limited. The aim of the study was to evaluate the frequency and predictors of disease reactivation in patients who stopped FTY for pregnancy. A multicentre retrospective cohort study was conducted in four Italian MS centres in 2013–2019. Both planned and unplanned pregnancies were included. The annualized relapse rate (ARR) was calculated before FTY treatment, during FTY treatment, during pregnancy and during the year after delivery. In total, 27 patients (mean age 29 years) were included. The ARR 1 year before FTY treatment was 1.3. Patients were exposed to FTY for a median of 2.9 years. The ARR was 0.04 during the last year before conception (*p* < 0.001 compared with the ARR before FTY treatment). Eleven patients became pregnant after a mean of 88 days following FTY discontinuation, whereas 16 patients stopped FTY after pregnancy confirmation. Relapses were observed in 22% of patients during pregnancy and in 44% in the postpartum period. ARR increased both during pregnancy (0.49; p = 0.027) and in the first year after delivery (0.67; *p* < 0.001) compared to the last year before pregnancy. Compared with radiological assessment before pregnancy, more patients showed new or enlarging T2 lesions (63% vs 30%; *p* = 0.02) and gadolinium-enhancing lesions (44% vs 0; *p* = 0.0001) on brain Magnetic Resonance Imaging. Relapses during pregnancy were the only significant predictor for postpartum relapses (OR 1.9, 95% CI 1.11–3.1). One case of spontaneous abortion and no cases of abnormal foetal development were observed. Despite adequate and prolonged control of disease activity, women who discontinue FTY because of pregnancy are at risk for disease reactivation. In patients who relapsed during pregnancy, the initiation of high-efficacy disease modifying drugs (DMDs) soon after delivery is advisable to prevent postpartum relapses.

**Supplementary Information:**

The online version contains supplementary material available at 10.1007/s13311-021-01106-6.

## Introduction

Multiple sclerosis (MS) affects 2.8 million people worldwide [1] and is considered the most prevalent cause of disability in young adults, resulting in physical, cognitive and psychosocial impairments [2]. MS is an immune-mediated inflammatory disorder of the central nervous system characterized by relapses caused by a new or enlarging demyelinating plaque [3]. Evidence suggests that early initiation of effective disease-modifying drugs (DMDs) leads to better outcomes in relapsing remitting MS patients (RRMS), reducing relapse rates and preventing disease progression [4]. MS is most prevalent in women of reproductive age [5, 6]; thus, pregnancy issues associated with new treatments are highly relevant. After being diagnosed with MS, at least 20 to 30% of women will have children [7, 8]. Previous studies on the course of MS during pregnancy were performed in patients either not exposed or minimally exposed to DMDs before pregnancy [9]. This situation does not accurately reflect current clinical practice, in which greater than 80% of patients with early-stage RRMS receive DMDs [10]. Furthermore, up to 30% of pregnancies are unplanned; thus, embryonal DMD exposure is relatively common in the first weeks of gestation [11]. Many DMDs and symptomatic treatments used in MS are not considered completely safe in women who are attempting to conceive, are pregnant or are breastfeeding [12–14]. Moreover, in MS, withdrawal of certain DMDs, mainly lymphocyte antitrafficking therapies, such as natalizumab and fingolimod (FTY), may result in severe disease reactivation or even rebound of disease activity. Thus, it is extremely important to properly evaluate the risk behind discontinuation or early pregnancy exposure [15, 16]. There is no univocal definition of disease rebound in MS, but rebound is generally accepted as the occurrence of new severe neurologic symptoms together with a significant increase in new or enlarging T2-weighted or gadolinium-enhancing (Gd +) T1-weighted lesions exceeding baseline activity after treatment discontinuation [17]. This unpredictable disease reactivation can be severe and potentially disabling and is particularly of concern to women on FTY treatment who are planning a pregnancy given the drug washout period. Although pregnancy has classically been associated with a significant reduction in the clinical relapse rate, there are several reports of dramatic disease reactivation during pregnancy following withdrawal of FTY treatment [16, 18–27]. Recent studies estimated an incidence of 4–25% of disease rebound after withdrawal of FTY for any reason [28], but specific data on the disease reactivation rate after FTY withdrawal due to pregnancy (planned or unplanned) are limited. It is important to have predictors of disease reactivation risk after discontinuation of FTY that could be used to counsel patients who plan to become pregnant. The aim of the study was to evaluate the frequency and predictors of disease reactivation in a multicentric retrospective cohort of patients with MS who stopped FTY for pregnancy planning or after early accidental exposure in unplanned patients.

## Patients and Methods

### Study Design and Patient Cohort

This multicentre retrospective cohort study was conducted in four Italian MS centres in 2013–2019. The inclusion criteria were as follows: diagnosis of RRMS according to the McDonald criteria [29], previous treatment with FTY for at least 12 consecutive months, FTY withdrawal due to pregnancy planning or accidental exposure in unplanned pregnancy. Patients who had bridging therapy for more than 6 months prior to conception were excluded. FTY was prescribed according to the criteria of the Italian Medicines Agency (AIFA); briefly, all subjects started FTY due to aggressive disease from onset (naïve patients), inefficacy of first-line treatments or a high risk of progressive multifocal leukoencephalopathy during natalizumab therapy (switching patients).

Data were retrieved from clinical charts and included patient demographics, MS onset, disease duration, previous treatments, duration of FTY treatment and clinical and radiological activity 1 and 2 years prior to starting FTY therapy, during FTY treatment, in the last year before pregnancy, during pregnancy and 1 year after delivery. A relapse was defined as the appearance or reappearance of one or more symptoms attributable to MS accompanied by objective deterioration as shown by neurologic examination, lasting at least 24 h, in the absence of fever and preceded by neurologic stability for at least 30 days [30]. The annualized relapse rate (ARR) was calculated before FTY treatment, during FTY treatment, during each trimester of pregnancy and during the year after delivery. Magnetic resonance imaging (MRI) data were analysed when at least one MRI study in the year before pregnancy and during the 6 months from childbirth was available. Disability was assessed using the Expanded Disability Status Scale (EDSS) [31] 1 year prior to FTY cessation, at FTY withdrawal and after delivery. Lymphocyte counts before drug discontinuation were also collected. The type of delivery (vaginal or caesarean), newborn outcomes and breastfeeding history and DMD resumption after delivery were retrieved. All patients provided written informed consent to include their anonymized data in the study.

### Statistical Analysis

Demographics and clinical and radiological characteristics are expressed as the mean and standard deviation (SD) or median and interquartile range (IRQ) for continuous variables or absolute frequency and percentages for categorical variables. Continuous variables were compared between groups with *t*-tests or non-parametric Mann–Whitney *U* tests as appropriate following variable distribution. Categorical variables were compared by using a chi-square test or two-tailed Fisher’s exact test, as appropriate. ARR comparisons were calculated by the Wilcoxon signed-rank-test. A 2-tailed value of *p* < 0.05 was considered significant. Statistical analyses were performed using Stata software (StataCorp. 2015. Stata Statistical Software: Release 14. College Station, TX: StataCorp LP).

## Results

In 2013–2019, 3787 patients with RRMS received one or more DMDs in the four study centres. Among these, 785 patients (21% of the whole cohort) were exposed to FTY. We identified a total of 27 women (3% of all patients treated with FTY in the whole cohort) who stopped FTY treatment for planned or unplanned pregnancy.

### Patient Characteristics Before Pregnancy

**Fig. 1:**
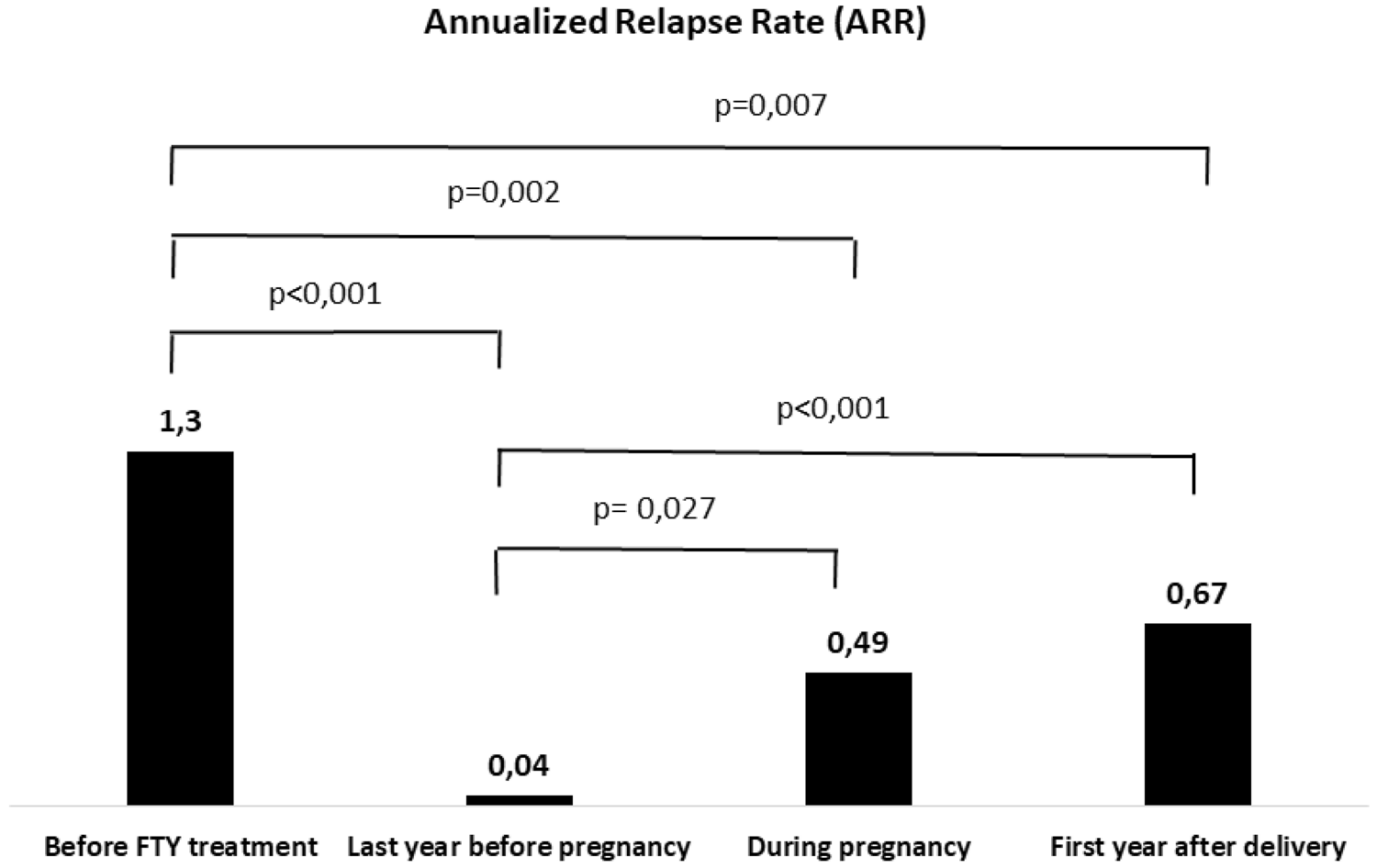
Annualized relapse rate (ARR) in the whole cohort 1 year before fingolimod (FTY) treatment, in the last year before pregnancy, during pregnancy and in the first year after delivery. Approximately 15% of patients were FTY treatment naïve. Comparisons were performed using the Mann–Whitney test

Patient characteristics before pregnancy are described in Table 1. The median age was 29 years, the disease duration at FTY discontinuation was 9.1 years, and the EDSS score was 2.0. Most patients (74%) started FTY treatment after switching from first-line therapies. In contrast, 11% switched from natalizumab due to a high risk of progressive multifocal leukoencephalopathy, and 15% were naïve to DMDs with an aggressive disease course. The ARR before FTY treatment was 1.3. Patients were exposed to FTY for a median of 2.9 years. The ARR was 0.2 during the entire FTY treatment and 0.04 during the last year of FTY treatment (both *p* < 0.001 compared with ARR before FTY treatment: Fig. 1). Only one patient (3.7%) experienced one relapse in the year before pregnancy. In the last year before pregnancy, 30% of patients showed new or enlarging T2 lesions on brain MRI, which were limited to 1 or 2 lesions in most cases. None of the patients showed gadolinium-enhancing lesions in the year before pregnancy.

**Table 1:** Patient characteristics before pregnancy

Women	*n* = 27
Age, years, median (IQR)	29.0 (25–33)
BMI, kg/m^2^, mean (SD)	22.6 (3.2)
Disease duration at FTY discontinuation, years, median (IQR)	9.1 (4.3–13.7)
EDSS score, median (IQR)	2.0 (1.0–3.5)
Last DMD before FTY, *n* (%)	
No previous treatment Interferon beta-1a Glatiramer acetate Natalizumab	4 (14.8%) 15 (55.6%) 5 (18.5%) 3 (11.1%)
ARR one year before FTY treatment, mean (95% CI)	1.3 (1.0–1.5)
FTY exposure, years, median (IQR)	2.3 (1.2–4.1)
ARR on FTY, mean (95% CI)	0.2 (0.1–0.4)
ARR Last year on FTY treatment, mean (95% CI)	0.04 (0–0.11)
Patients relapsing in the year before pregnancy, (%)	1 (3.7%)
No. of T2 lesions on brain MRI during last year on FTY treatment, mean (SD)^a^	0.5 (1.0)
No. of T2 lesions on brain MRI during last year on FTY treatment, *n* (%)	
0 1 2 3	19 (70.4%) 3 (11.1%) 3 (11.1%) 2 (7.4%)
Time from FTY discontinuation to pregnancy confirmation, days, mean (SD)	29.3 (67.4)

*ARR* annualized relapse rate, *BMI* body mass index, *CI* confidence interval, *DMD* disease-modifying drug, *EDSS* Expanded Disability Status Score, *FTY* fingolimod, *IQR* interquartile range, *MRI* magnetic resonance imaging, *SD* standard deviation

^a^Including new or enlarging T2 lesions; no gadolinium-enhancing lesions were observed

### Clinical Relapses During Pregnancy and After Delivery

During pregnancy, ten relapses in six (22%) patients were recorded (Table 2; Fig. 1). One patient experienced three relapses, two patients experienced two relapses, and three patients experienced one relapse. The mean ARR during the entire pregnancy period was significantly greater than the mean ARR reported last year before pregnancy [0.49 (95% CI 0.08–0.91) vs 0.04 (95% CI 0–0.11) *p* = 0.027] but lower than the mean ARR reported before FTY treatment [0.49 (95% CI 0.08–0.91) vs 1.3 (95% CI 1.0–1.5), *p* = 0.002)]. During the first trimester of pregnancy, six relapses in four patients were reported. Two patients experienced two relapses, and two patients experienced one relapse. The mean ARR during the first trimester was not significantly greater than the mean ARR reported last year before pregnancy [0.89 (95% CI 0–1.80) vs 0.04 (95% CI 0–0.11), *p* = 0.077] but lower than the mean ARR reported before FTY treatment [0.89 (95% CI 0–1.80) vs 1.3 (95% CI 1.0–1.5), *p* = 0.048]. During the second trimester of pregnancy, four relapses in four patients were reported. Two patients also experienced relapses in the first trimester. The mean ARR during the second trimester was not significantly greater than the mean ARR reported last year before pregnancy [0.59 (95% CI 0.02–1.17) vs 0.04 (95% CI 0–0.11), *p* = 0.066] but was significantly lower than the mean ARR reported before FTY treatment [0.59 (95% CI 0.02–1.17) vs 1.3 (95% CI 1.0–1.5), *p* = 0.022] and comparable with the mean ARR reported in the first trimester of pregnancy [0.59 (95% CI 0.02–1.17) vs 0.89 (95% CI 0–1.80), *p* = 0.480]. No relapses occurred in the third trimester.

**Table 2:** ARR during FTY, during pregnancy, and after delivery

Women	*n* = 27	*p*-value^a^
Last year on FTY treatment	0.04 (0–0.11)	–
Pregnancy First trimester Second trimester Third trimester	0.49 (0.08–0.91) 0.89 (0–1.80) 0.59 (0.02–1.17) 0	0.027 0.077 0.066 –
Post delivery 0–6 months 6–12 months	0.67 (0.38–0.96) 1.03 (0.53–1.55) 0.30 (0.01–0.58)	< 0.001 0.001 0.066
Combined pregnancy and 12 months post-delivery	0.62 (0.32–0.93)	0.001

*ARR* annualized relapse rate, *FTY* fingolimod

^a^Compared to the relapse rate during the year before pregnancy, calculated using the Wilcoxon signed-rank-test. Data are expressed as the mean (95% CI)

In the first year after delivery, 18 relapses in 14 (44%) patients were recorded (Table 2; Fig. 1). Four patients experienced two relapses, and ten patients experienced one relapse. All six patients who relapsed during pregnancy experienced at least one relapse in the year after delivery. The median time to first postpartum relapse was 40 days (± 107.6). The mean ARR in the first year after delivery was significantly greater than the mean ARR reported last year before pregnancy [0.67 (95% CI 0.38–0.96) vs 0.04 (95% CI 0–0.11), *p* < 0.001] but lower than the mean ARR reported before FTY treatment [0.67 (95% CI 0.38–0.96) vs 1.3 (95% CI 1.0–1.5), *p* = 0.007)] and comparable with the mean ARR during the entire pregnancy period [0.67 (95% CI 0.38–0.96) vs 0.49 (95% CI 0.08–0.91), *p* = 0.183]. Splitting the postdelivery year into two periods, 14 relapses in 12 patients were recorded in the first 6 months. Two patients experienced two relapses, and ten patients experienced one relapse. Eleven patients relapsed in the first 3 months postpartum. Five patients who relapsed during pregnancy experienced at least one relapse in the first 6 months after delivery. The mean ARR in the first 6 months after delivery was significantly greater than the mean ARR reported last year before pregnancy [1.03 (95% CI 0.53–1.55) vs 0.04 (95% CI 0–0.11), *p* = 0.001], comparable with the mean ARR reported before FTY treatment [1.03 (95% CI 0.53–1.55) vs 1.3 (95% CI 1.0–1.5), *p* = 0.344] and not significantly higher than the mean ARR during the entire pregnancy period [1.03 (95% CI 0.53–1.55) vs 0.49 (95% CI 0.08–0.91), p = 0.056]. In the second semester, four relapses in four patients were recorded. Two patients also experienced relapse in the first semester, and one patient relapsed during pregnancy. The mean ARR in the second semester after delivery was not significantly greater than the mean ARR reported last year before pregnancy [0.30 (95% CI 0.01–0.58) vs 0.04 (95% CI 0–0.11), *p* = 0.066], was significantly lower than the mean ARR reported before FTY treatment [0.30 (95% CI 0.01–0.58) vs 1.3 (95% CI 1.0–1.5), *p* < 0.001], and was comparable with the mean ARR during the entire pregnancy period [0.30 (95% CI 0.01–0.58) vs 0.49 (95% CI 0.08–0.91), *p* = 0.395].

Combining both the pregnancy period and first year after delivery, 28 relapses in 14 (44%) patients were recorded (Table 2). The mean ARR in the combined period was significantly higher than the mean ARR reported last year before pregnancy [0.62 (0.32–0.93 95% C.I.) vs 0.04 (0–0.11 95% C.I.), *p* = 0.001] but significantly lower than the mean ARR reported before FTY treatment [0.62 (0.32–0.93 95% C.I.) vs 1.3 (1.0–1.5 95% C.I.), *p* = 0.002]. In our cohort, eight patients (30%) experienced an increased ARR compared to the ARR reported before FTY treatment.

### Radiological Activity After Delivery

During pregnancy, only two patients underwent MRI examination for severe relapses. After delivery, all patients underwent MRI examination at a median of 68.2 days from delivery to evaluate radiological disease reactivation. Compared with radiological assessment before pregnancy, more patients showed new or enlarging T2 lesions on brain MRI (63% vs 30%, *p* = 0.02), and more patients showed three or more T2 lesions (37% vs 7.4%, *p* = 0.010). Furthermore, 44% of patients exhibited gadolinium-enhancing lesions compared with no patients in the pre-pregnancy period (*p* = 0.0001).

Comparing relapsing versus non-relapsing patients, new or enlarging T2 lesions on MRI were more frequent in relapsing patients, and more relapsing patients showed three or more T2 lesions than non-relapsing patients (Table 3). Similarly, gadolinium-enhancing lesions were more frequent in relapsing patients, and more relapsing patients showed three or more Gd + lesions than non-relapsing patients (Table 3). The time from delivery to MRI was not significantly different between relapsing and non-relapsing patients.

**Table 3:** Magnetic resonance imaging activity after delivery

	**Women, *n* = 27**	**Relapsing patients, *n* = 12**	**Non-relapsing patients, *n* = 15**	***p*-value**
New/enlarging T2 lesions	17 (63.0)	11 (91.7)	6 (30.0)	**0.006**
Number of T2 lesions				
0–2	17 (63.0)	3 (25.0)	14 (93.3)	**< 0.001**
≥ 3	10 (37.0)	9 (75.0)	1 (6.7)	
Any Gd + lesions	12 (44.4)	8 (66.7)	4 (33.3)	**0.004**
Number of GD + lesions				**0.030**
0–2	21 (77.8)	7 (58.3)	14 (93.3)	
≥ 3	6 (22.2)	5 (41.7)	1 (6.7)	
Time from delivery to first MRI, median (SD)	68.2 (37.6)	58.9 (31.2)	75.7 (41.5)	0.258

All values are reported as numbers (percentages) unless indicated otherwise

*Gd* + gadolinium enhancing, *MRI* magnetic resonance imaging, *SD* standard deviation

### Clinical and Radiological Outcomes in Planned and Unplanned Pregnancies

Given that pregnancy planning potentially exposes patients to a longer washout period from FTY than unplanned pregnancies, we conducted a subanalysis in the two patient cohorts. Pregnancy was planned in 11 patients (41%) with a mean wash-out period from FTY discontinuation to pregnancy of 88.0 days; no relapses were observed during the wash-out period. Clinical and radiological patient characteristics are described in supplementary Table 1. Patients who planned pregnancy were younger and had shorter disease duration and a lower EDSS score, but no differences were observed in terms of relapse rate during pregnancy or after delivery or radiological outcomes.

### Predictors of Disease Reactivation

Table 4 summarizes possible predictors of disease reactivation during pregnancy. Neither demographics (age, BMI), clinical characteristics (disease duration, EDSS score, naïve status, ARR before FTY treatment and before pregnancy, duration of FTY exposure), MRI activity, lymphocyte count or time from FTY suspension to pregnancy confirmation were significantly associated with disease reactivation during pregnancy.

**Table 4:** Predictors of disease reactivation during pregnancy

	**Relapsing patients,** ***n* = 6**	**Non-relapsing patients, *n* = 21**	***p* value**
Age, years	25.8 (5.8)	31.1 (6.0)	0.068
Disease duration at FTY start, years	4.0 (2.8)	7.7 (6.0)	0.166
EDSS score	2.6 (1.6)	2.1 (1.6)	0.555
BMI, kg/m^2^	22.4 (2.4)	22.7 (3.5)	0.899
Naïve, *n* (%)	0	4 (19.0)	0.247
ARR 1 year before FTY	1.5 (0.8)	1.2 (0.6)	0.317
FTY exposure, years	2.6 (2.0)	3.0 (2.4)	0.751
Relapse last year on FTY	0	0.1 (0.5)	0.477
MRI activity last year on FTY, *n* (%)	1 (16.7)	7 (33.0)	0.430
Last lymphocyte count on FTY, cell/mm^3^	515 (110)	570 (180)	0.466
Time from FTY suspension to pregnancy confirmation, days	70.1 (118.9)	17.4 (41.4)	0.088

All values are reported as the mean (standard deviation) unless indicated otherwise

*ARR* annualized relapse rate, *BMI* body mass index, *EDSS* Expanded Disability Status Score, *FTY* fingolimod, *MRI* magnetic resonance imaging

Table 5 describes predictors of disease reactivation after delivery. Notably, disease reactivation during pregnancy was significantly associated with disease reactivation after delivery (OR 1.9; 95% CI 1.11–3.1; *p* = 0.030).

**Table 5:** Predictors of disease reactivation after delivery

	**Relapsing patients, *n* = 12**	**Non-relapsing patients, *n* = 15**	***p*-value**
Age, years	29.9 (8.0)	30.0 (4.9)	0.974
Disease duration at FTY start, years	7.3 (6.5)	6.5 (5.4)	0.700
EDSS score	2.3 (1.8)	2.2 (1.4)	0.884
BMI, kg/m^2^	22.9 (3.8)	22.4 (2.9)	0.684
Naïve, *n* (%)	0	4 (26.7)	0.053
ARR 1 year before FTY	1.2 (0.7)	1.3 (0.6)	0.522
FTY exposure, years	3.4 (2.4)	2.5 (2.1)	0.296
Relapse last year on FTY	0.1 (0.3)	0.1 (0.5)	0.767
MRI activity last year on FTY, *n* (%)	5 (41.7)	3 (20.0)	0.221
Last lymphocyte count on FTY, cell/mm^3^	580 (170)	550 (180)	0.724
Time from FTY suspension to pregnancy confirmation, days	46.8 (87.9)	15.3 (43.6)	0.235
**Relapses during pregnancy, *n* (%)**	**5 (41.7)**	**1 (6.7)**	**0.030**
Time from delivery to DMD initiation, days	120.1 (143.4)	142.6 (130.3)	0.686

All values are reported as the mean (standard deviation) unless indicated otherwise

*ARR* annualized relapse rate, *BMI* body mass index, *DMD* disease-modifying drugs, *EDSS* Expanded Disability Status Score, *FTY* fingolimod, *MRI* magnetic resonance imaging

### Pregnancy Outcome

The pregnancy outcome was characterized by 26 live birth babies and one spontaneous abortion at 10 weeks of gestation that occurred in the setting of a planned pregnancy with an 8-week wash-out period prior to conception. The mean gestational age was 38.2 weeks (2.4). Of 16 unplanned pregnancies (59%) with in utero exposure to FTY, no cases of foetal death occurred. No cases of abnormal foetal development were observed in either planned or unplanned pregnancies. Caesarean delivery was performed in 14 patients (54%). Breastfeeding was chosen by 23 patients (88%) for a median of 3 months.

After delivery, twenty-one patients (77.8%) resumed FTY treatment, whereas six patients (22.2%) switched to another treatment (natalizumab in two patients, ocrelizumab in two patients, alemtuzumab in one patient and dimethyl fumarate in one patient). Treatment was resumed after a mean of 132.5 ± 134.2 days following delivery.

## Discussion

In our study, we observed significant disease reactivation during pregnancy (mostly in the first trimester) and after delivery (mostly in the first semester) in women with MS, showing good disease control before FTY discontinuation due to pregnancy. In this peculiar clinical setting, disease activity in pregnant MS patients depends on a trade-off between the effect of pregnancy on disease activity and the discontinuation of highly effective but teratogenic DMDs.

According to the previous experience of Confavreux et al., in patients minimally exposed to DMDs, ARR declines during pregnancy, especially in the third trimester, and increases during the first 3 months postpartum before returning to the pre-pregnancy rate. This condition is associated with an oestrogen-driven shift away from cell-mediated immunity towards increased humoral immunity[9]. In women with MS, secretion of cytokines, such as interleukin-10, activin-A and programmed death ligand-1 (PD-L1), induces a tolerogenic status towards the foeto-placental unit and likewise suppresses MS activity [32]. In pregnant women affected by MS, the tolerogenic status can also be mediated by selective expansion of CD4( +)CD25( +)Foxp3( +) T regulatory cells [33].

In recent years, pregnant MS patient population characteristics have changed with the widespread use of DMDs. Many patients with highly active MS, well controlled with highly effective DMDs, started to plan pregnancies, although many DMDs are not recommended during pregnancy due to their teratogenic effects. Therefore, in recent years, an increase in relapses during pregnancy compared to the pre-pregnancy period has been more frequently observed and is probably due to the withdrawal of highly effective DMDs before conception [16]. For FTY, a 2-month washout period is required when planning pregnancy before attempting conception [34]. According to pregnancy risk information reported by the Food and Drug Administration, FTY may cause foetal harm based on animal data because it can diffuse across the placenta and bind to receptors responsible for vascular system formation [35]. A total of 66 pregnancies with in utero exposure to FTY were collected from phase II, III and IV clinical trials, reporting 5 cases (7.6%) of abnormal foetal development [36]. The European Medicines Agency has estimated that FTY is associated with a twofold increased risk of severe congenital malformations (especially cardiovascular, renal and musculoskeletal malformations) when administered during pregnancy [37]. In a recent Swiss study, elective termination of pregnancy in pregnant women affected by MS occurred twice as often in patients exposed in the first trimester to FTY compared to those exposed to beta-interferon [38].

Unfortunately, the washout period and the unpredictable time to pregnancy confirmation can expose MS patients to a high risk of disease reactivation. The onset of pregnancy does not always compensate for disease reactivation due to withdrawal of DMDs [39]. As observed in the cohort of pregnant MS patients described by Alroughani et al., ARR was significantly increased during pregnancy compared to the pre-pregnancy year on FTY therapy (0.29 vs 0.03), especially in patients with longer wash-out periods [16]. In our cohort, relapses primarily occurred in the first trimester probably due to the washout period from FTY. Conversely, in the second trimester and particularly in the third trimester, the tolerogenic effect of pregnancy is prominent and decreases the relapse rate.

It is unclear whether the disease reactivation observed in our study represents a rebound syndrome. Rebound syndrome is a clinical condition that emerges after discontinuation of “antitrafficking” treatments, such as natalizumab and FTY. Mechanisms of rebound after FTY withdrawal include B cell reconstitution; a rapid influx and increase in self-reactive T cells, particularly central memory T cells, the activation of antibody production by T cells and a decrease in direct S1P receptor-mediated activity on astrocytes, oligodendrocytes and neurons [40, 41]. Specific diagnostic criteria for FTY rebound syndrome remain limited, and four different definitions have been proposed in the literature with the common feature of a level of recurrent disease activity exceeding that observed prior to starting FTY treatment. In 2016, Hatcher et al. published a single-centre retrospective cohort study including 46 patients and proposed the following rebound definition: “new severe neurological symptoms after ceasing fingolimod treatment (within 4–16 weeks) with the development of multiple new Gd-enhancing T1 lesions exceeding baseline activity” [18]. Afterwards, Frau et al. performed a multicentre retrospective cohort study including 100 patients and used a purely clinical definition of rebound, namely, a relapse with EDSS increase ≥ 2 or ≥ 2 relapses in the 6 months following FTY discontinuation and never experienced in the patient’s lifetime [25]. In a post hoc analysis of post-study discontinuation rebound in FREEDOMS and FREEDOMS II trials, including 402 patients treated with 0.5 mg/day FTY, two different rebound definitions were proposed. The first definition was exclusively clinical based and considered rebound as one or more severe relapses within 7 months after FTY discontinuation. The second definition was exclusively radiologically based and considered rebound as a Gd-enhancing T1 lesion volume greater than the 95% upper prediction limit within 3 months after FTY discontinuation [42]. Finally, in a single-centre retrospective cohort study including 31 patients, Uygunoglu et al. proposed a new rebound definition that combines clinical, radiological and disability progression outcomes; in particular, the authors defined rebound as > 5 Gd-enhanced lesions and/or tumefactive demyelinating lesions on MRI and clinical and MRI activity worse than pre-FTY treatment and increased by at least 1 point on EDSS [43]. Unfortunately, a shared definition of rebound does not currently exist, and there are no elements in favour of one specific definition. Given this variability in definitions, the rebound rates after FTY withdrawal range from 3.7% in the Vermersch study [42] to 25% of patients in the Uygunoglu study [43] over a maximum follow-up period of 7 months with an average time to relapse varying from 7.6 to 15 weeks after FTY discontinuation. No specific data on rebound after FTY withdrawal in the pregnancy-associated setting have been reported. In our study, 30% of patients experienced an increased ARR compared to that reported before FTY treatment, likely representing a rebound. However, it is necessary to achieve consensus on an unambiguous definition of rebound before evaluating the incidence rates and its putative predictors. We chose disease reactivation, which is a more objective and shared parameter, as the outcome for our analysis of predictors after FTY withdrawal due to pregnancy. Based on the analysis of demographic, clinical and radiological features, lymphocyte count and time from FTY suspension to pregnancy confirmation, it was not possible to detect significant prognostic factors for disease reactivation. However, patients who relapsed during pregnancy also had an increased risk for relapse in the postpartum period. Our findings are consistent with the cohort of 156 patients who discontinued FTY due to pregnancy presented by Haemat et al., showing that the incidence of relapse during pregnancy in 25% of patients with relapses during pregnancy was the only significant predictor for relapses postpartum [44]. Given that most of the postpartum relapses occurred in the first 3 months in our cohort, in our opinion, restarting DMD treatment as soon as possible is recommended in these patients, even if this treatment would impact breastfeeding.

The limitations of this study include the small sample size and the retrospective design. However, this study provides valuable information that can be useful when considering pregnancy planning in patients treated with FTY or in managing unplanned pregnancies during FTY treatment.

In conclusion, clinicians should alert women with MS on FTY treatment who are planning to conceive about the possibility of increased disease activity after FTY discontinuation, even if adequate and prolonged control of disease activity is achieved with therapy. Women who discontinue FTY to become pregnant need to be closely monitored given the risk of disease reactivation. The possibility of bridging to a safer drug, such as interferon beta, glatiramer acetate or natalizumab, can be evaluated in individual patients. In patients who relapsed during pregnancy, the initiation of high-efficacy DMDs soon after delivery is advisable to prevent postpartum relapses.

## Supplementary Information

Below is the link to the electronic supplementary material.

Supplementary file1 (PDF 465 KB)

Supplementary file2 (PDF 491 KB)

Supplementary file3 (PDF 468 KB)

Supplementary file4 (PDF 465 KB)

Supplementary file5 (PDF 468 KB)

Supplementary file6 (PDF 480 KB)

Supplementary file7 (PDF 471 KB)

Supplementary file8 (PDF 468 KB)

Supplementary file9 (DOCX 18 KB)
