# Supplementary material for: Disease Reactivation after Fingolimod Discontinuation in Pregnant Multiple Sclerosis Patients
Source: Neurotherapeutics. 2021 Sep 7;18(4):2598–607. doi: 10.1007/s13311-021-01106-6 (PMC8803993; doi:10.1007/s13311-021-01106-6)
Supplement: Supplementary file 9 — Supplementary file9 (DOCX 18 KB) [file 13311_2021_1106_MOESM9_ESM.docx]

**Supplementary table 1.** Planned and unplanned pregnancies: clinical outcomes during and after pregnancy and radiological outcomes after delivery.

|  | **Planned**  **pregnancies,**  **n=11** | **Unplanned pregnancies, n=16** | **p value** |
| --- | --- | --- | --- |
| Age, years, median (IQR) | 27 (24-32) | 30 (28-37.8) | **0.048** |
| Disease duration at FTY start, years, median (IQR) | 4 (0-6) | 8.5 (4-12.5) | **0.004** |
| EDSS score, median (IQR) | 1.5 (1.0-2.0) | 2.0 (1.5-4.0) | **0.007** |
| BMI, Kg/m2 | 23.1 (3.8) | 22.2 (2.8) | 0.504 |
| Naïve, n (%) | 2 (18.2) | 2 (12.5) | 0.683 |
| ARR one year before FTY | 1.3 (0.8) | 1.3 (0.6) | 0.933 |
| FTY exposure, years | 3.4 (2.2) | 2.6 (2.4) | 0.368 |
| Relapse last year on FTY | 0 | 0.1 (0.3) | 0.333 |
| MRI activity last year on FTY, n (%) | 5 (45.5) | 3 (18.8) | 0.135 |
| Relapsing patients during pregnancy, n (%) | 4 (36.4) | 2 (12.5) | 0.143 |
| ARR during pregnancy | 1.1 (1.5) | 0.2 (0.5) | 0.251 |
| ARR one year after delivery | 0.7 (0.6) | 0.6 (0.8) | 0.718 |
| New/enlarging T2 lesions | 7 (63.6) | 10 (62.5) | 0.952 |
| Number of T2 lesions  0-2  ≥3 | 6 (54.5)  5 (45.5) | 11 (68.7)  5 (31.3) | 0.453 |
| Any Gd+ lesions | 6 (54.5) | 6 (37.5) | 0.381 |
| Number of GD+ lesions  0-2  ≥3 | 7 (63.6)  4 (36.4) | 14 (87.5)  2 (12.5) | 0.143 |
| Time from delivery to first MRI | 63.0 (37.9) | 71.8 (38.2) | 0.560 |

*All values are reported as mean (standard deviation) unless indicated otherwise. ARR: Annualized Relapse Rate; BMI: Body Mass Index; EDSS: Expanded Disability Status Score; FTY: fingolimod; MRI: Magnetic Resonance Imaging; Gd+: Gadolinium enhancing.*
